# Genomic characterization and prognostic significance of copy number alterations in Tunisian patients with acute lymphoblastic leukemia

**DOI:** 10.1371/journal.pone.0340696

**Published:** 2026-02-03

**Authors:** Ameni Bedoui, Wajdi Ayadi, Nour Louati, Imen Frikha, Yosra Fakhfakh, Fahmi Smaoui, Ali Gargouri, Ikram Ben Amor, Moez Elloumi, Moez Mdhaffar, Raja Mokdad Gargouri

**Affiliations:** 1 Laboratory of Molecular Biotechnology of Eukaryotes, Center of Biotechnology of Sfax, University of Sfax, Sfax, Tunisia; 2 Regional Center of Blood Transfusion of Sfax-LR19SP05, University of Sfax, Sfax, Tunisia; 3 Faculty of Medicine of Sfax, University of Sfax, Sfax, Tunisia; 4 Department of Clinical Hematology, Hedi Chaker University-Hospital, Sfax, Tunisia; 5 Laboratory of Microbiology, Research Laboratory for Microorganisms and Human Disease LR03SP03, Habib Bourguiba University-Hospital, Sfax, Tunisia; Hirosaki University Graduate School of Medicine, JAPAN

## Abstract

Acute lymphoblastic leukemia (ALL) is a heterogeneous malignancy characterized by various genomic alterations playing a crucial role in disease classification, prognosis, and response to treatment. However, molecular diagnosis and effective management of this hematological malignancy remain a major challenge, particularly in developing countries, including Tunisia. In this study, we aimed to conduct a detailed analysis of copy number alterations (CNAs) associated with ALL in a cohort of 60 primary samples from Tunisian patients. Using multiplex ligation-dependent probe amplification (MLPA), major genetic lesions, including *IKZF1*, *CDKN2A/2B*, *PAX5*, *ETV6*, *BTG1,* and genes located in the *PAR1* region, were analyzed and their associations with clinical and laboratory features, as well as survival outcomes, were also evaluated. Our analysis revealed that 70% of patients had deletions and/or amplifications in at least one gene. The most frequently observed deletions were in *CDKN2A/2B* (33.3%, n = 20), *IKZF1* (30%, n = 18), and *PAX5* genes (25%, n = 15). *BTG1* deletions were significantly associated with female gender, *IKZF1* deletions were more frequent in adult patients, in those with elevated white blood cell (WBC) counts, and in cases involving the *BCR::ABL1* translocation, while duplications of the *PAR1* region were significantly associated with hyperdiploïdy. Regarding treatment response, cases of *IKZF1* deletions showed a significant association with poor glucocorticoid response (GC) at day 8 of treatment and positive minimal residual disease (MRD) rates at days 33 and 63, particularly in B-ALL cases. Furthermore, patients with *IKZF1* deletions were associated with significantly lower survival rates in both univariate and multivariate analyses compared to those without these deletions. Additionally, the integration of *IKZF1* deletion status into risk stratification models revealed markedly different survival outcomes, highlighting its potential interest in developing new stratification algorithms. These results underscore the critical importance of molecular profiling, particularly *IKZF1* status, for improving outcomes in ALL patients in Tunisia.

## Introduction

Acute lymphoblastic leukemia (ALL) is a malignancy of B- or T-lineage lymphoblast’s, characterized by the abnormal clonal expansion of immature progenitor cells in the bone marrow (BM). These leukemic cells can infiltrate the peripheral blood (PB) and disseminate to other organs, including the central nervous system, lymph nodes, liver, and spleen [1–3]. ALL is a common childhood malignancy, accounting for up to 25% of childhood cancers. It can also occur in adulthood, with variable clinical, biological, and molecular features across age groups [4,5]. The incidence of ALL varies considerably among countries [6]. Globally, the estimated age-standardized incidence rate increased from 1.23 per 100,000 in 1990 to 1.96 in 2019, with a marked rise in cases among older individuals, particularly in populations with a higher socio-demographic index [7]. According to the latest Tunisian Cancer Registry, ALL accounts for 45.1% of all hematological malignancies, with a standardized incidence rate of 2.25. B-ALL is the most common subtype, representing 71% of cases, of which 78% occur in pediatric/young adult patients. In contrast, T-ALL accounts for 29% of cases, a proportion that appears higher than that reported in European populations [8]. Over the past few decades, the management of ALL has evolved considerably, leading to notable improvements in survival and cure rates, particularly among pediatric patients. This progress is mainly attributable to major advances in molecular diagnostics, risk stratification, and the approval of targeted therapies [9]. Despite these advancements, relapse, which affects approximately 15–20% of patients, remains a major clinical challenge, particularly in low- and middle-income countries, where access to advanced diagnostics and therapies is often limited [10].

Although the exact etiology of ALL is still not fully understood, ongoing research continues to elucidate the genetic abnormalities that impair hematopoiesis and promote leukemogenesis. Interestingly, several genetic alterations have emerged as valuable prognostic and therapeutic markers, playing a critical role in risk stratification and guiding appropriate treatment strategies. Adverse prognostic indicators include fusion genes such as *BCR::ABL1* and *MLL::AFF1*, which are associated with a high risk of relapse and poorer outcomes [11–13]. In contrast, favorable prognostic markers such as *ETV6::RUNX1* and high hyperdiploïdy are correlated with better treatment responses and long-term survival [14,15].

More recently, the use of next-generation sequencing (NGS) and multiplex ligation-dependent probe amplification (MLPA) has significantly advanced our understanding of molecular pathogenesis, defining a broad spectrum of genetic abnormalities, including CNAs, particularly micro-deletions in genes involved in B-lymphocyte development [16–18]. One of the most clinically relevant genes is *IKZF1* (Ikaros family zinc finger 1), which encodes a transcription factor essential for the development and function of lymphocytes, especially B-cell precursors [19,20]. *IKZF1* plays a pivotal role in regulating the immune system by controlling the differentiation and activity of various immune cell types, including B- and T-lymphocytes [19–21].

Besides *IKZF1*, particular attention has been given to CNAs affecting other genes, including *ETV6*, *PAX5*, and *EBF1*, essential for B-cell differentiation, as well as *CDKN2A/2B*, *BTG1*, and *RB1*, involved in cell cycle regulation and tumor suppression [4,18,22,23]. Furthermore, CNAs disrupting cytokine receptor genes such as *CRLF2, IL3RA* and *CSF2RA* located in the pseudoautosomal region 1 (*PAR1)* region have also been identified to be associated with aberrant activation of cytokine-mediated signaling pathways, which play a critical role in leukemogenesis [24,25]. In fact, *CRLF2* gene overexpression often results from *PAR1* rearrangements, such as *IGH::CRLF2* translocations or *P2RY8::CRLF2* fusions, leading to constitutive activation of the *JAK-STAT* signaling pathway, which promotes leukemic cell proliferation and survival [24–26]. Although most of these gene alterations demonstrate prognostic relevance in ALL patients, deletions in *IKZF1* have emerged as particularly clinically significant [27,28]. This has led to the integration of *IKZF1* status into modern risk stratification schemes and highlighted its utility in identifying patients who might benefit from intensified therapeutic approaches [29,30]. However, the optimal treatment strategy for ALL patients with *IKZF1* deletions remains controversial, with ongoing debate regarding the use of *IKZF1* deletion status as a basis for high-risk stratification.

To support these advances, the present study aimed to provide a detailed analysis of the broader spectrum of genetic alterations associated with ALL, as well as clinical relevance and prognostic significance in Tunisian patients. Such valuable information could support the continued monitoring of progress in risk stratification criteria, with a view to more tailored and individualized therapeutic decision-making.

## Materials and methods

### Study cohort

Bone marrow and/or peripheral blood samples containing more than 60% blasts were retrospectively collected from 60 patients newly diagnosed with ALL at the Hemobiology Laboratory of the Regional Blood Transfusion Center in Sfax, Tunisia, between January 2020 and March 2024. Samples were obtained as part of routine clinical procedures for biological diagnosis, and only the residual material not required for diagnostic purposes was used in the present study. Patients’ ages ranged from 1 to 72 years; 40 were under 30 (pediatric/young adult form) and 20 were over 30 (adult form). The diagnosis of precursor cell origin was based on the conventional FAB (French–American–British) classification and immunophenotypic criteria; 45 patients (75%) were diagnosed with B-cell precursor ALL, and 15 patients (25%) with T-cell precursor ALL. The main characteristics and genetic features of the study cohort are shown in Table 1. Ethical approval was granted by the local Ethics Committee of the Faculty of Medicine in Sfax (approval no. 12/25, dated February 14, 2025). The samples were fully anonymized before being accessed on February 15, 2025, for research purposes. The Ethics Committee waived the requirement for informed consent because no identifiable information about participants was available after data collection.

**Table 1:** Clinical and genetic features of the ALL studied cases (n = 60).

	Total (N = 60)	B-ALL (n = 45)	T-ALL (n = 15)
**Characteristics**			
**Gender**			
**Male**	35	23	12
**Female**	25	22	3
**Age, year**			
**Median (range),**	14 (0.5-70)	18.5 (0.5-70)	9.5 (3-62)
**WBC count,.10** ^**3**^ **/µl**			
**Median, range**	11.2 (0.9-59.30)	9.75 (0.9-59.30)	21 (2.1-44.30)
**Immunophenotype**			
**B-ALL**			
**Pro B**	4	4	–
**Pre-B**	6	6	–
**Common B**	22	22	–
**B-other**	13	13	–
**T-ALL**			
**Early T**	1	–	1
**Pro**	4	–	4
**Common T**	4	–	5
**T-other**	5	–	5
**Cytogenetic (Gene fusion)**			
**BCR::ABL**	12	12	0
**MLL::AF4**	2	2	0
**TCF::PBX1**	1	1	0
**TEL::AML1**	1	1	0
**Cytogenetic (Karyotype)**			
**Hyperdiploïdy**	10	9	1
**Others**	50	36	14
**Protocol risk group: EORTC**			
**Standard**	32	26	6
**High**	8	2	6
**Protocol risk group: GRALL**			
**Standard**	1	1	–
**High**	19	16	3
**Flow cytometry-MRD Status:**			
**MRD day 33 Positive (≥10**^**−2**^)	29	21	8
**MRD day 63 Positive (≥10**^**−3**^)	32	25	7
**Event**			
**Death**	23	19	4
**Relapse**	34	26	8
**Late**	7	6	1
**Early**	27	20	7

As internal controls for methodological standardization of the MLPA assay, 15 samples from healthy individuals were collected between December 2020 and March 2023, following written informed consent. The control samples were obtained from volunteer blood donors with no prior clinical history or relevant medical conditions, referred to the Hemobiology Laboratory of the Regional Blood Transfusion Center in Sfax, Tunisia.

### Therapy groups and risk stratification

Patients were treated at the Clinical Hematology Department of the Hedi Chaker University Hospital in Sfax, Tunisia, according to the EORTC-CLG 58951 protocol (European Organization for Research and Treatment of Cancer), developed for pediatric and young adult patients, or the GRALL-2014 protocol (Adult Acute Lymphoblastic Leukemia Research Group), designed for the management of adult patients over 30 years old. Philadelphia chromosome-positive (Ph+) patients were treated according to the GRAAPH-2005 protocol, which combines a tyrosine kinase inhibitor (TKI) with intensive chemotherapy. Minimal Residual Disease (MRD) analysis was performed by flow cytometry (FCM) on a Becton Dickinson FACS Canto II® cytometer. Follow-up bone marrow samples were collected on days 33 and 63, with a minimum of 100,000 events acquired per sample.

Based on the following criteria: normal bone marrow morphology (<5% blasts and >25% cellularity), an absolute neutrophil count >1.5 × 10³/µL, a platelet count >100 × 10³/µL, and resolution of all extramedullary disease, complete remission (CR) was achieved in 56 patients (93.3%), while the remaining patients experienced treatment failure with >5% blasts cells. Initial risk stratification was performed based on age, WBC count at diagnosis, blast cell counts at day 8, and key cytogenetic alterations, including *BCR::ABL1* and *ETV6::RUNX1* fusions, *MLL* rearrangements, hyperdiploidy, and hypodiploidy. For pediatric cases, based on these criteria, 8 cases were classified as high risk (HR) and the remaining 32 cases as standard risk (SR). Among adult patients, 19 were assigned to the high-risk group (Table 1).

### DNA extraction

Blood and bone marrow (BM) samples were digested in a proteinase K lysis buffer containing 100 μg/mL proteinase K, 5 mM NaCl, 50 mM Tris–HCl (pH 7.5), 0.5% SDS, and 10 mM EDTA, then incubated at 56 °C for 2–4 hours with constant agitation. After digestion, DNA was extracted with phenol/chloroform/isoamyl alcohol mixture (25:24:1) and precipitated with absolute ethanol. The DNA pellet was collected, washed with 70% ethanol, air-dried, and rehydrated in sterile water. The quality and quantity of the extracted DNA were assessed with a NanoDrop® 2000 spectrophotometer (Thermo Scientific). DNA samples were stored at −20 °C until used for downstream PCR and MLPA analyses.

### MLPA analysis

To identify CNAs in ALL samples, MLPA was performed using the SALSA P335-C2 kit (MRC Holland, Amsterdam, Netherlands), following the manufacturer’s instructions. The P335 probe mix allows for the detection of deletions and duplications in genes involved in lymphocyte differentiation and cell cycle regulation (*IKZF1, CDKN2A/B, PAX5, EBF1, ETV6, BTG1*, and *RB1*), as well as in genes located in the *PAR1* of chromosomes X and Y (*CRLF2, CSF2RA, SHOX, IL3RA, and P2RY8*). These target genes and chromosomal regions are known to play an important diagnostic or prognostic role in ALL. They were selected after a thorough literature review and based on recommendations from experts in ALL research. The probemix also contains 13 reference probes serving as internal controls for data normalization. In addition to the patient samples, ten healthy control DNA samples were included for inter-sample normalization. MLPA steps, including DNA denaturation, probe hybridization, ligation, and PCR amplification, were performed in a 96-well PCR thermocycler (BioGener Thermal Cycler). PCR products were separated by capillary electrophoresis on an ABI 3500 Prism Genetic Analyzer (Applied Biosystems). MLPA data were analyzed using Coffalyser.Net™ software (MRC Holland) with default settings, which allowed for intra-sample and inter-sample normalization using the manufacturer’s internal reference probes and the healthy control samples, respectively. Peak heights from each sample were compared to those of healthy donor controls to calculate relative ratio values. A cut-off ratio of ≥ 1.3 was used to define duplications, while a ratio of ≤ 0.75 indicated deletions. Alterations in *CDKN2A* and/or *CDKN2B* were combined and reported as *CDKN2A/2B* abnormalities. Similarly, an alteration in at least one gene in the *PAR1* region (*CRLF2, CSF2RA, IL3RA, or SHOX*) was classified as a *PAR1* abnormality. Additionally, the *IKZF1*^*plus*^ profile was defined based on the report of stanulla. et al [31]. Briefly, the patient was considered *IKZF1*^*plus*^ positive if deletions of the *IKZF1* gene coexisted with deletions of *CDKN2A/CDKN2B, PAX5*, and/or *PAR1* region.

### Multiplex PCR for ERG gene deletions

To further investigate cases meeting the *IKZF1*^*plus*^ profile criteria, and as *ERG* has been shown to mitigate the poor prognosis associated with *IKZF1* deletions, *ERG* gene deletion analysis was performed by multiplex PCR, following previously established primers and PCR conditions [32]. As an internal control, an additional forward primer located in exon 11 of the *ERG* gene (ERG11F: cagagggctccttcaaacacag) was included in the PCR reactions, which generate a 620 bp control fragment [32].

### Statistical analyses

The statistical analyses focused exclusively on gene deletions; gene duplications were excluded due to their low frequency, except for the PAR1 region, where only duplications were considered. Data were analyzed using SPSS software (version 22.0). Differences between categorical variables were compared using the chi-square test or Fisher’s exact test, as appropriate. Survival distributions were analyzed using the Kaplan–Meier method and compared using the log-rank test. Overall survival (OS) was calculated from the date of diagnosis to death or last follow-up. Relapse-free survival (RFS) was calculated from CR to the date of first relapse. The Cox proportional hazards model was used to obtain the estimate and the 95% confidence interval (CI) of the hazard ratio (HR) of the instantaneous event rate in one group versus another. Univariate and multivariate Cox hazard models were used to determine independent prognostic predictors. Multivariable analyses were performed to investigate the independent impact of *IKZF1* on OS and RFS using Cox regression models, which are based on age (≥ vs. < 30 years), *BCR::ABL1* (presence vs. absence), diploid karyotype (hyperdiploïdy vs. other status), WBC (≥ vs. < 50.10^3^/µl), MRD day-33 (≥ vs. < 10^-2^), MRD day-63 (≥ vs. < 10^-3^), Initial risk stratification and treatment protocol (GRALL-2014 trial vs. EORTC-CLG 58951 trial). Variables associated with p < 0.05 were considered statistically significant. CNA status was analyzed dichotomously as the deleted versus non-deleted (excluding duplication), without distinction between different isoforms.

## Results

### Copy number alteration frequencies in Tunisian ALL cases

A total of 130 CNAs, including intragenic deletions (n = 89) and duplications (n = 51), were identified in 42 out of 60 (70%) patients with ALL. Notably, 71.4% of these cases (n = 30) had at least two combined CNAs. Deletions were generally more frequent than duplications (72% vs. 28%), except in genes within the *PAR1* region, which were predominantly duplicated (16.6%, n = 10/60). The most frequently detected deletions, either alone or in combination, concerned the *CDKN2A*/2B (33.3%, n = 20), *IKZF1* (30%, n = 18), and *PAX5* genes (25%, n = 15). Focusing on the spectrum of *IKZF1* gene alterations, whole-gene deletions were mainly seen in 8 of 18 deleted cases (44.4%), followed by the ∆4–7 deletion (IK6 variant), which produces the dominant-negative isoform (n = 5; 29.4%). The ∆2–7 and ∆4–8 deletions were each found in 2 cases (11.7%), while the ∆2–8 deletion was present in only 1 case (5.8%), illustrating the diversity of *IKZF1* deletion profiles. Regarding the duplication in the *PAR1* region, whole-gene duplication was observed in 6 cases, while 4 patients harbored partial duplications, mainly affecting the *CSF2RA* gene.

Since *ERG* gene deletions were not detected in any of the 60 analyzed cases, the co-occurrence of *IKZF1* deletions with deletions in *PAX5*, *CDKN2A/2B*, and/or *PAR1* allowed the identification of an *IKZF1*^*plus*^ profile, as defined by Stanulla et al. (2018), in 10 out of the 18 patients with *IKZF1* deletions (55.5%). It was detected in co-occurrence with *PAX5* and *CDKN2A/2B* deletions in 5 cases, with *PAX5* deletions alone in 3 cases, and with *CDKN2A/2B* deletions alone in the remaining 2 cases.

### CNA frequencies according to baseline characteristics

**Fig 1:**
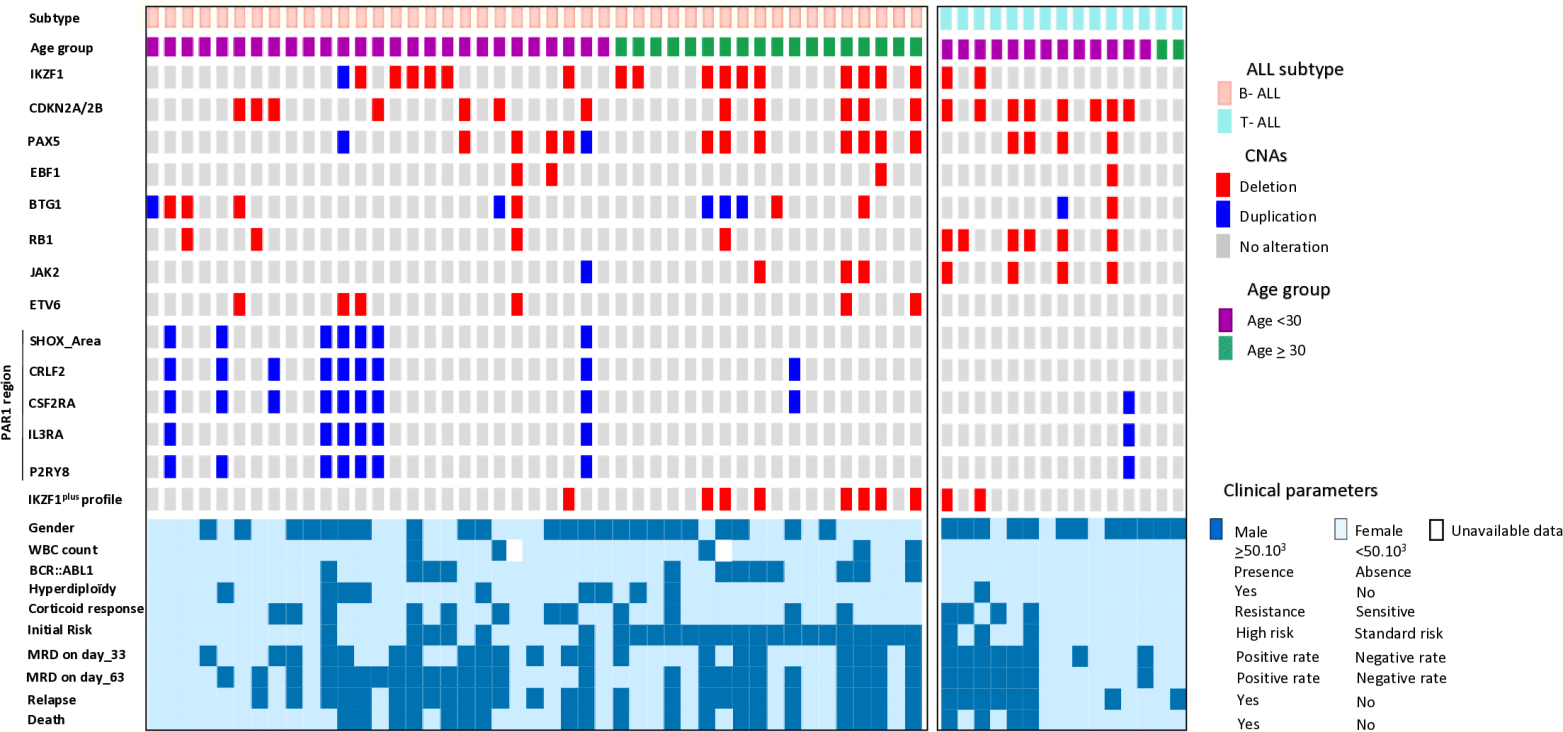
Oncoprint illustrating the molecular characterization of the CNA spectrum across ALL subtypes and clinical parameters in Tunisian patients (n = 60). Oncoplot presents the landscape of CNAs detected by the MLPA P335 panel, alongside corresponding clinical features for each patient.

The presence or absence of recurrent CNAs, including deletions in *IKZF1*, *PAX5*, *CDKN2A/2B*, *JAK2*, and *BTG*; duplications in the *PAR1* region; as well as the *IKZF1*^*plus*^ profile, was assessed based on the baseline characteristics of Tunisian ALL patients (Fig 1). Our analysis revealed that *IKZF1* deletions and the *IKZF1*^*plus*^ profile, defined by the co-occurrence of *IKZF1* deletions with deletions in genes such as *PAX5* or *CDKN2A/2B,* were significantly associated with adult ALL patients (p = 0.034 and p = 0.012, respectively) and with a high WBC count at diagnosis (p = 0.023 for both), as well as the presence of a *BCR::ABL1* translocation (p < 0.001 and p = 0.021, respectively). Furthermore, *BTG* deletions were significantly associated with female gender (p = 0.007), while *PAR1* region duplications were significantly associated with hyperdiploïdy (p = 0.008) (Table 2).

**Table 2:** CNA status according to patient characteristics and response to treatment in the entire group (n = 60).

	Total, n	Gender		Age		WBC count*		BCR::ABL1		Diploidy		Corticoid response		MRD_33		MRD_63		Risk classification			Relapse		Death	
		M	F	<30	>30	<50.10^3^	>50.10^3^	Yes	No	Hyper	Other	R	S	P	N	P	N		HR	SR	Yes	No	Yes	No
***IKZF1* gene**																								
**Deleted**	18	9	9	8	10	13	4	10	8	2	16	8	10	13	5	16	2		14	4	8	0	16	2
**Non-deleted**	42	26	16	32	10	40	1	2	40	8	34	8	34	16	26	16	26		13	39	16	26	7	35
		*p*=0.409		***p=*0.034**		***p=*0.023**		***p<*0.0001**		*p=*0.708		*p*=0.058		***p=*0.024**		***p=*0.001**		***p=*0.001**			***p<*0.0001**		***p<*0.0001**	
***IKZF1***^***Plus***^ **profile**																								
**Presence**	10	4	6	3	7	6	3	5	5	0	10	4	6	9	1	9	1		8	2	10	0	9	1
**Absence**	50	31	19	37	13	47	2	7	43	10	40	12	38	20	30	23	27		19	31	24	26	14	36
		*p*=0.294		***p=*0.012**		***p=*0.023**		***p=*0.021**		*p=*0.188		*p*=0.433		***p=*0.005**		***p=*0.014**		***p=*0.033**			***p=*0.003**		***p<*0.001**	
***CDKN2A*/2B gene**																								
**Deleted**	20	11	9	15	5	16	3	5	15	2	18	5	15	13	7	15	5		8	12	14	6	9	11
**Non-deleted**	40	24	16	25	15	37	2	7	33	8	32	11	29	16	24	17	23		19	21	20	20	14	26
		*p*=0.785		*p*=0.395		*p=*0.318		*p=*0.511		*p=*0.471		*p*=1		*p=*0.100		***p=*0.027**		*p=*0.784			*p=*0.174		p=0.575	
***PAX5* gene**																								
**Delete**	15	8	7	8	7	10	3	5	10	1	14	5	10	11	4	10	5		8	7	12	3	9	6
**Non-deleted**	45	27	18	23	13	43	2	7	38	9	36	11	34	18	27	22	23		19	26	22	23	14	31
		*p*=0.765		*p*=0.223		*p=*0.069		*p=*0.153		*p=*0.426		*p*=0.516		***p=*0.037**		*p=*0.371		*p=*0.554			***p=*0.041**		p=0.067	
***EBF1* gene**																								
**Deleted**	4	2	2	3	1	3	0	0	4	0	4	2	2	3	1	2	2		1	3	2	2	1	3
**Non-deleted**	56	33	23	37	19	50	5	12	44	10	46	14	42	26	30	30	26		26	30	32	24	22	34
		*p*=1		*p*=1		*p=*1		*p=*0.574		*p=*1		*p*=0.287		*p=0.346*		*p=*1			*p=*0.620		*p=*1		p=1	
***BTG1* gene**																								
**Deleted**	7	1	6	5	2	5	1	2	5	0	7	1	6	3	4	3	4		2	5	3	4	1	6
**Non-deleted**	53	34	19	35	18	48	4	10	43	10	43	15	38	26	27	29	24		25	28	31	22	22	31
		***p*=0.017**		*p*=1		*p=*0.433		*p=*0.619		*p=*0.589		*p*=0.663		*p=1*		*p=*0.695			*p=*0.442		*p=*0.454		p=0.233	
***RB1***																								
**Deleted**	10	7	3	9	1	8	0	1	9	1	9	3	7	6	4	6	4		2	8	7	3	2	8
**Non-deleted**	50	28	22	31	19	45	5	11	39	9	41	13	37	23	27	23	24		25	25	27	23	21	28
		*p*=0.499		*p*=0.142		*p=*1		*p=*0.670		*p=*1		*p=*1		*p=*0.500		*p*=0.737			*p=*0.162		*p=*0.491		p=0.291	
***ETV6***																								
**Deleted**	6	2	4	4	2	4	1	2	4	2	4	2	4	4	2	5	1		2	4	6	0	4	2
**Non-deleted**	54	33	21	36	18	49	4	10	44	8	46	14	40	25	29	27	27		25	29	28	26	19	35
		*p*=0.223		*p*=1		*p=*0.374		*p=*0.590		*p=*0.259		*p*=0.653		*p=*0.417		*p=*0.201			*p=*0.681		***p=*0.**031		p=0.191	
***JAK2***																								
**Deleted**	7	4	3	4	3	6	1	3	4	0	7	2	5	5	2	5	2		3	4	5	2	4	3
**Non-deleted**	53	31	22	36	17	47	4	9	44	10	43	14	39	24	29	27	26		24	29	29	24	19	34
		*p*=1		*p*=0.676		*p=*0.487		*p=*0.135		*p=*0.589		*p*=1		*p=*0.247		*p=*0.432			*p=*1		*p=*0.688		p=0.412	
***PAR1* region**																								
**Deleted**	11	6	4	9	1	10	0	1	9	5	5	1	9	4	6	7	3		3	7	5	5	4	6
**Non-deleted**	49	29	11	31	19	43	5	11	39	5	45	15	35	25	25	25	25		24	26	29	21	19	31
		*p*=1		*p*=0.142		*p=*0.575		*p=*0.670		***p=*0.008**		*p*=0.263		*p=*0.732		*p=*0.312			*p=*0.488		*p=*0.733		p=1	

* two cases remained undefined; M: Male; F: Female, L: low; H: High, R: Resistance; S: Sensitivity, P: Positive; N: Negative, HR: High risk; SR: Standard risk.

When analyzing the B-ALL subtype alone, these correlations were further confirmed. Moreover, other associations were observed, particularly the frequency of *PAR1* region duplications and *JAK2* gene deletions with pediatric/young adult cases (p = 0.06 and p = 0.05, respectively), as well as the significant association of *JAK2* gene deletions with *BCR::ABL1*-positive cases (p = 0.016) (S1 Table). Regarding the T-ALL group, no significant associations were observed with the previous baseline characteristics. In contrast, comparison of CNAs between B-ALL and T-ALL cases revealed a significantly higher frequency of *RB1* gene deletions in the T-ALL group (p = 0.011).

To further characterize the genomic landscape within each age subgroup, our analysis revealed that *IKZF1* deletions remained significantly associated with the *BCR::ABL1* translocation in both the pediatric/young adult and adult subgroups (p = 0.021 and p = 0.020, respectively). Age-specific associations were also identified, most notably the significant association between duplications in the *PAR1* region and a hyperdiploid karyotype, observed exclusively among pediatric/young adult patients (p = 0.008), (S2 and S3 Tables).

### CNAs’ status correlation with primary treatment response

To further investigate the clinical relevance of CNAs, we analyzed their association with primary treatment response, including corticosteroid response at day 8 and MRD status at days 33 and 63. Among the CNAs examined, *IKZF1* deletions showed a significant association with corticosteroid resistance, particularly within the B-ALL subgroup (p = 0.037) and among pediatric/young adult patients (p = 0.025). In the overall cohort, deletions affecting this key hematopoietic regulatory factor were also consistently associated with positive MRD during the induction phase on both day 33 and day 63 (p = 0.024 and p < 0.001). Additional significant associations were identified, particularly those involving alterations that co-occur with *IKZF1* deletions, *PAX5* and *CDKN2A/2B* gene deletions which define the *IKZF1*^*plus*^ profile. Similar trends were observed in subgroup analyses, particularly among adult patients and within the B-ALL subtype (Table 2, S1-S3 Tables).

### CNAs’ survival rates and outcomes

**Fig 2:**
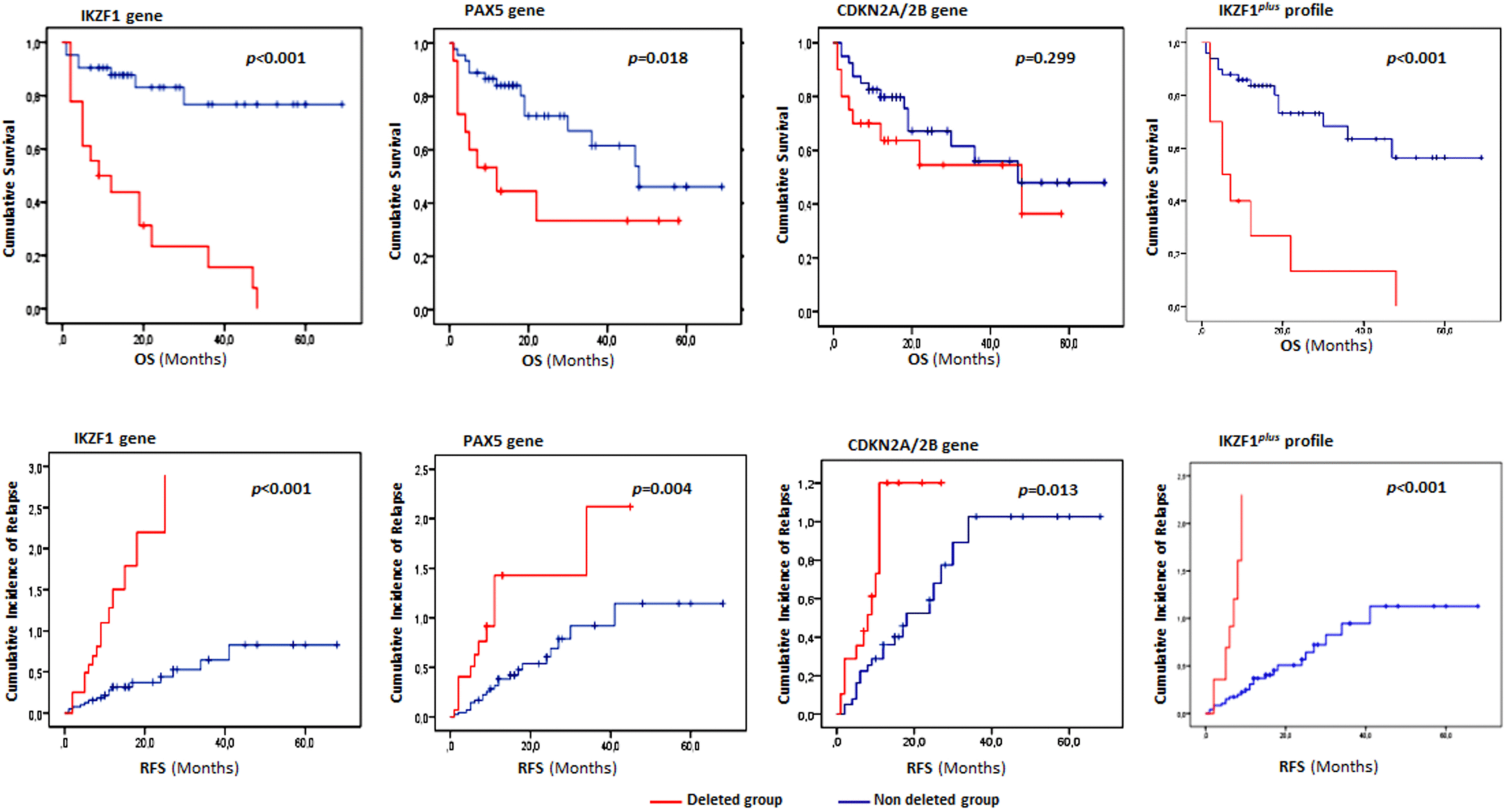
Overall survival and cumulative incidence of relapse in Tunisian ALL patients according to CNA status (deletion vs. no deletion). Red Kaplan–Meier curves represent patients harboring specific gene deletions, while blue curves correspond to wild-type cases. OS: Overall survival, RFS: relapse free survival.

The impact of gene deletions on survival, taken separately and in combined within the *IKZF1plus* profile was assessed using the Kaplan–Meier method. We showed that *IKZF1* and *PAX5* deletions were significantly associated with poor survival outcomes in the overall cohort. Notably, patients with *IKZF1* deletions had significantly lower outcomes in both RFS and OS than non-deletion carriers (p < 0.001). Additionally, patients with CDKN2A/2B deletions had shorter RFS than patients with the wild-type form (p = 0.006). Importantly, cases classified according to the *IKZF1*^*plus*^ profile also exhibit significantly poor survival outcomes with short OS and RFS rates (p < 0.0001), thus reinforcing its prognostic relevance (Fig 2).

When evaluating survival outcomes within each subgroup, these associations remained statistically significant in the B-ALL subtype, where *JAK2* deletions were also correlated with shorter OS (p = 0.021) and reduced RFS (p = 0.028) (S1 Fig). Furthermore, patients carrying *IKZF1* deletions or the *IKZF1*^*plus*^ profile consistently showed poorer OS and RFS in both pediatric/young adult and adult groups. Deletions affecting *PAX5* and *CDKN2A*/2B were likewise associated with inferior survival (p = 0.005 and p = 0.021, respectively), especially in adult patients (S2 and S3 Figs).

To assess whether *IKZF1* deletions and the *IKZF1*^*plus*^ profile retained prognostic significance independent of other clinical and molecular variables, a multivariate Cox regression model was performed. The model included the following covariates: age, *BCR::ABL1* translocation status, diploidy, WBC count at diagnosis, MRD status at days 33 and 63, and treatment protocols. Our results demonstrate that *IKZF1* deletions are independently associated with a significantly increased risk of death. In the overall cohort, patients harboring these deletions had a markedly worse OS (HR = 3.51; p = 0.027), with the effect being even more pronounced in the B-ALL subtype (HR = 6.66; p = 0.009) and in adult patients (HR = 9.16; p = 0.01). These findings highlight *IKZF1* deletions as a robust independent predictor of poor prognosis. Notably, MRD levels at days 33 and/or 63 remained statistically significant predictors of survival, underscoring their continued value as a key prognostic marker (Table 3, S4 and S6 Tables). In contrast, the *IKZF1*^*plus*^ profile was significantly associated with OS and RFS in univariate analyses, these associations were not maintained in multivariate models, suggesting that its prognostic impact may be influenced by other factors.

**Table 3:** Multivariate Cox model assessing the impact of *IKZF1* deletions on survival in ALL patients (n = 60).

	OS				RFS			
	*p* value	HR	CI 95%		*p* value	HR	CI 95%	
Parameters			Low	High			Low	High
***IKZF1* deletion**	**0.027**	3.514	1.155	10.688	0.078	2.417	0.906	6.448
**Age**	0.078	3.132	0.878	11.171	0.619	1.292	0.472	3.539
**BCR::ABL1**	0.380	0.577	0.169	1.970	0.217	0.498	0.165	1.507
**Diploidy**	0.142	2.710	0.717	10.248	0.458	0.683	0.250	1.868
**WBC count**	0.572	0.693	0.195	2.471	0.883	0.918	0.294	2.861
**MRD at day 33**	**0.014**	4.370	1.449	28.310	**0.029**	2.921	1.116	7.649
**MRD at day 63**	0.091	6.404	0.790	24.176	**0.002**	7.372	2.075	26.188
**Initial risk stratification**	0.615	1.391	0.384	5.033	0.280	1.861	0.604	5.737
**Treatment Protocol**	0.078	3.132	0.878	11.171	0.619	1.292	0.472	3.539

OS: Overall survival, RFS: Relapse free survival, HR: Hazard ratio, CI 95%: confidence interval 95%.

### Risk stratification refinement based on the *IKZF1* gene status

To further investigate the crucial prognostic value of *IKZF1* deletions, we analyzed their combination with established prognostic factors; Model 1: including initial risk stratification and Model 2: in combination with MRD status, assessing their impact on OS, RFS, and CIR. Based on this integrated approach, all patients in our cohort were reclassified into three molecular risk groups by combining *IKZF1* status with initial risk stratification: Standard molecular risk (initial standard-risk and wild-type *IKZF1*), High molecular risk (initial high-risk and wild-type *IKZF1*) and Very high molecular risk (initial standard- or high-risk with *IKZF1* deletion).

This redefined classification revealed a significant prognostic impact, with patients in the very high molecular risk group having significantly lower 5-year OS rates (10.5% vs. 61.5% vs. 96.4%, p < 0.001), compared with the high and standard molecular risk groups. There was also a statistically significant difference in OS between standard-risk with *IKZF1* deletion and cases in the same groups with wild-type *IKZF1* gene (96% vs. 21%, p < 0.001). These findings highlight the critical importance of incorporating molecular profiling, particularly early *IKZF1* deletion status, into risk stratification to improve prognostic accuracy and guide more effective therapeutic decision-making.

**Fig 3:**
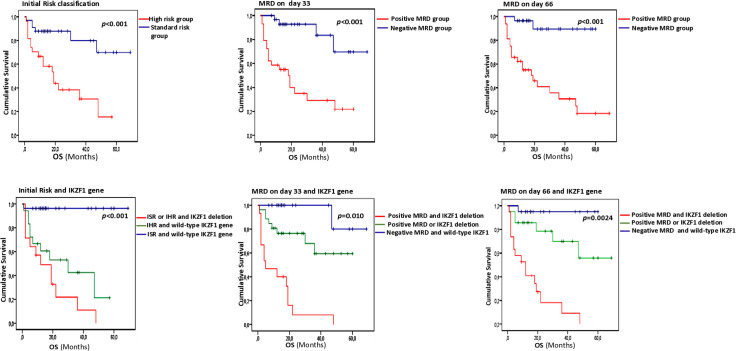
Overall survival of ALL patients according to the new risk stratification (n = 60). Patients were stratified into Model 1: Standard molecular risk (initial standard-risk and wild-type *IKZF1*), High molecular risk (initial high-risk and wild-type *IKZF1*) and Very high molecular risk (initial standard- or high-risk with *IKZF1* deletion); Model 2: Standard (MRD-negative and *IKZF1* wild-type), intermediate (MRD-positive or *IKZF1* deletion), and high-risk (MRD-positive and *IKZF1* deletion) groups.

A second redefined model recently described by Deng et al. (2025) was based on MRD status and *IKZF1* gene profile, stratifying patients into Standard (MRD-negative and *IKZF1* wild-type), intermediate (MRD-positive or *IKZF1* deletion), and high-risk (MRD-positive and *IKZF1* deletion) groups. Similarly, the standard risk group was strongly associated with higher 5-year OS rates than the intermediate and high-risk groups. This result confirmed the low impact of the *IKZF1* gene deletion rather within the standard risk group (Fig 3). No statistically significant results were noted for the RFS level.

## Discussion

Our study is the first to assess the frequency, heterogeneity, and clinical significance of CNAs in an unselected cohort of both pediatric/young adult and adult ALL Tunisian patients. Although CNAs are well established as essential prognostic markers in contemporary treatment protocols, they are not routinely assessed in our hospital settings [5,8,33,34]. Therefore, this study was designed to characterize the CNAs profile in the Tunisian ALL population, to identify specific genetic signatures that could guide future risk-adapted therapeutic strategies and ultimately enhance clinical outcomes.

To achieve this aim, we used the MLPA technique, a reliable and efficient molecular method capable of detecting a broad spectrum of CNAs, including *IKZF1* deletions and other common gene abnormalities in ALL. MLPA offers a practical and scalable approach for large-scale clinical screening and research, making it particularly suitable for implementation even in resource-limited settings, thus improving accessibility to essential genetic diagnostics. In our analysis, 70% of the ALL cases studied had genetic abnormalities, with the most frequent deletions observed in *CDKN2A/2B* (33.3%), *IKZF1* (30%), and *PAX5* (25%), while deletions in *EBF1* (6.7%) and *ETV6* (10%) were comparatively less frequent. Overall, the CNA distribution is consistent with previous results established among children and adult patients using the MLPA method, highlighting the increasing clinical relevance of CNA profiling in patient management. However, a literature review reveals considerable variability in reported CNA frequencies, likely due to differences in study design, particularly the selective inclusion of specific entities, such as pediatric versus adult-onset, and T-ALL versus B-ALL subtypes [4,34–35]. A major conclusion drawn from these observations is that ALL patients exhibit distinct genetic landscapes, reflecting fundamental biological differences that significantly influence clinical outcomes and therapeutic responses [36–39]. In fact, our study revealed a notable age-related variation in *IKZF1* gene deletions, with a significantly higher frequency observed in adult patients (50%) compared to pediatric cases (20%). Furthermore, we observed that *IKZF1* gene deletions were more frequent in patients with *BCR::ABL1* translocation in the B-ALL subtype (p = 0.001). Our findings are consistent with previously reported trends, reinforcing the role of *IKZF*1 deletions, which often co-occur with high-risk factors such as *BCR::ABL1* transcript, in contributing to increased disease aggressiveness, particularly among adult B-cell patients [36,40–43]. Compared with adult B-ALL patients, pediatric/young adult cases had a lower frequency of deletions and a higher incidence of duplications, especially in genes within the *PAR1* region. Moreover, these gene duplications were significantly associated with hyperdiploïd karyotype, a cytogenetic feature historically linked to favorable prognosis in ALL [44,45]. Among these genes, the *CRLF2* gene has received a particular attention due to its recurrent association with genetic abnormalities, leading to elevated expression that appears to define a distinct subgroup of B-ALL and offers potential molecular targets for treatment [46–48]. Although these abnormalities often include *P2RY8::CRLF2* fusions resulting from interstitial deletions, our results revealed no such rearrangements [26]. Instead, we identified the presence of additional copies of the *CRLF2* locus in 9 cases, probably due to supernumerary X chromosomes, which could also contribute to *CRLF2* overexpression [24,49,50]. Even though duplications in the *PAR1* region are not frequently documented, similar findings have been reported by Schmäh et al. (2017). They showed that high *CRLF2* expression, associated with increased gene copy number, is typically characterized by rare additional deletions and hyperdiploidy karyotypes, in contrast to *P2RY8::CRLF2* fusion and *IGH::CRLF2* translocation cases, which are associated with additional deletions. Regarding the CNA frequencies within the B and T immunophenotypic groups, our results revealed uneven distributions, particularly among *RB1* gene deletions, which were significantly more frequent in T-ALL cases, aligning with previous findings [51]. Although *RB1* is traditionally considered to play a crucial role in B-cell differentiation, emerging data suggest that its functional impact varies depending on the hematopoietic context. Indeed, most studies have associated *RB1* deletions with impaired differentiation and increased aggressiveness in B-lineage leukemia [52,53].

The second major challenge of our study was to assess the importance of the identification of CNAs, often described as a poor prognostic factor, in the treatment response and survival of Tunisian patients with T/B precursor ALL. In the early stages of treatment, glucocorticoid resistance represents a critical factor in ALL and is recognized as a major contributor to therapeutic failure and disease relapse. The resistant phenotype has been linked to multiple molecular drivers, including activation of the *AKT* and *ERK* signaling pathways, which are frequently triggered in *IKZF1*-deficient leukemic cells [54]. Furthermore, combined loss of *BTG1* and *IKZF1* has been shown to further increase glucocorticoid resistance [47]. In our cohort, all 16 ALL patients with a resistant phenotype had at least one CNA in *IKZF1*, *CDKN2A/2B*, and/or *PAX5* genes, with a statistically significant association observed specifically for *IKZF1* gene deletions, particularly among B-cell ALL cases (p = 0.037). Similar findings were reported by Braun et al. (2022) [55], who studied 373 children with B-cell precursor ALL and observed significantly lower glucocorticoid responses in patients with *IKZF1* deletions. Although the role of *CDKN2A/2B* deletions is still under investigation, other studies have concluded that patients with *CDKN2A/B* deletions were more frequently steroid-resistant and exhibited a higher risk of poor prednisolone response [22]. Additionally, these gene deletions have been shown to correlate with MRD positivity during various treatment assessments [22,56,57]. Consistent with these findings, our cohort also demonstrated a significant association between *IKZF1* deletions and MRD positivity, specifically on day-33 of induction therapy and day-63 following consolidation, in the overall ALL patients (p = 0.024 and p < 0.001, respectively), as well as within the B-ALL subtype (p = 0.035 and p = 0.002, respectively) and among adult patients (p = 0.005 and 0.003, respectively).

As expected, our findings showed that CNAs associated with primary treatment failure are also powerful prognostic markers, closely related to poor outcomes, as indicated by a significant reduction in OS as well as increased CIR. Interestingly, deletions in the transcription factor *IKZF1* have been widely described as an independent marker of poor prognosis in both pediatric and adult ALL [39,42,43]. Consistent with these data, our study showed that the presence of *IKZF1* gene deletions is a reliable and independent predictor of poor survival outcomes in ALL. This association was confirmed by multivariate analyses, revealing that patients harboring *IKZF1* deletions had a markedly worse OS in the overall cohort (HR = 3.51; p = 0.027), with an even stronger adverse impact observed in the B-ALL subtype (HR = 6.66; p = 0.009) and in adult patients (HR = 9.16; p = 0.01). Our results closely mirror previous research, which has consistently shown that *IKZF1* deletions are linked to inferior survival outcomes and an increased risk of relapse [39,45,58–60], underscoring the critical importance of *IKZF1* status in risk stratification and therapeutic decision-making in ALL.

To improve outcome prediction and refine risk assessment in ALL, the integration of *IKZF1* deletions into established prognostic factors, such as initial risk stratification and MRD, is increasingly proposed. This approach allows the identification of subgroups with variable outcomes, as *IKZF1* deletions are associated with poor prognosis even within the standard-risk group. Therefore, these patients can be considered for treatment intensification or alternative therapeutic strategies. Notably, studies by Waanders et al. (2011) and Deng et al. (2025) have demonstrated that the combination of MRD status and *IKZF1* deletions offers superior prognostic value than either factor alone. In line with these findings, our results showed that the highest 5-year OS rate was observed in patients with both MRD-negative status and wild-type *IKZF1*, reaching 96%. This rate was significantly higher than in patients with either negative MRD alone (70%) or *IKZF1* wild-type alone (78%). In addition, a redefined model based on initial risk and *IKZF1* deletion status revealed a significant prognostic impact, as patients in the very high molecular risk group exhibited markedly inferior 5-year OS rates, compared with high and standard molecular risk (p < 0.001), consistent with previous studies [43,61]. Such a refined approach to risk assessment could enable more personalized and effective treatment strategies for ALL, potentially reducing treatment-related toxicity and improving long-term outcomes.

The relatively small sample size decreases statistical power and may limit the generalizability of our findings. Additionally, the absence of complementary cytogenetic analyses and comprehensive molecular profiling restricts the full characterization of the genetic landscape and its clinical significance. The dataset’s limitations also limit the application of advanced statistical frameworks, such as the Moorman classification, which could offer deeper insights into the patterns and prognostic implications of concomitant CNAs. Therefore, future studies with larger, well-characterized cohorts and integrated genomic approaches are crucial to approve these results.

## Conclusion

Our study highlights the clinical importance of early detection of CNAs, including *IKZF1* deletion, using MLPA. Integrating this molecular approach into routine diagnostics should refine risk stratification, enable more accurate diagnosis, and guide treatment decisions. By identifying genetic alteration profiles early, clinicians can more effectively tailor therapeutic strategies, potentially improving patient outcomes.

## Supporting information

S1 Table Correlation of CNA frequencies with clinicopathological features and treatment responses in the B-ALL group (n = 45). (DOCX)

S2 Table Correlation of CNA frequencies with clinicopathological features and treatment responses in the ALL pediatric/young adult group (n = 40). (DOCX)

S3 Table Correlation of CNA frequencies with clinicopathological features and treatment responses in the ALL Adults group (n = 20). (DOCX)

S4 Table Multivariate Cox model assessing the impact of IKZF1 deletions on survival in B-ALL cases (n = 45). (DOCX)

S5 Table Multivariate Cox model assessing the impact of IKZF1 deletions on survival in the ALL pediatric/young adult group (n = 40). (DOCX)

S6 Table Multivariate Cox model assessing the impact of IKZF1 deletions on survival in the ALL adult group (n = 20). (DOCX)

S1 Fig Overall survival and cumulative incidence of relapse in the B-ALL subtype according to CNA status (deletion vs. no deletion). Red Kaplan–Meier curves indicate patients exhibiting CNA-associated gene deletions; blue curves represent wild-type (non-deleted) genotypes. OS: Overall survival, RFS: relapse free survival.
(TIF)

S2 Fig Overall survival and cumulative incidence of relapse in the Pediatric/Young adult ALL cases according to CNA status (deletion vs. wild-type). Kaplan–Meier curves illustrate OS and CIR stratified by the presence or absence of specific gene deletions. Red curves represent patients harboring CNA-associated deletions, whereas blue curves correspond to wild-type (non-deleted) cases. OS: Overall survival, RFS: relapse free survival.
(TIF)

S3 Fig Overall survival and cumulative incidence of relapse in adult ALL cases according to CNA status (deletion vs. wild-type). Kaplan–Meier curves correspond to OS and CIR stratified by the presence or absence of specific gene deletions in each adult case. Red curves represent patients harboring CNA-associated deletions, whereas blue curves correspond to wild-type (non-deleted) cases. OS: Overall survival, RFS: relapse free survival.
(TIF)
