# Supplementary material for: Genomic characterization and prognostic significance of copy number alterations in Tunisian patients with acute lymphoblastic leukemia
Source: PLoS One. 2026 Feb 3;21(2):e0340696. doi: 10.1371/journal.pone.0340696 (PMC12867238; doi:10.1371/journal.pone.0340696)
Supplement: S1 Table — (DOCX) [file pone.0340696.s001.docx]

**S1 Table. CNA status according to patient characteristics and response to treatment in the B-ALL group (n=45).**

|  | **Total, n** | **Gender**  **M F** | **Age**  **<30 >30** | **WBC count***  **<50.10^3^ >50.10^3^** | **BCR::ABL1**  **Yes No** | **Diploidy**  **Hyper Other** | **Corticoid response**  **R S** | **MRD_33**  **P N** | **MRD_63**  **P N** | **Risk classification**  **HR SR** | **Relapse**  **Yes No** | **Death**  **Yes No** |
| --- | --- | --- | --- | --- | --- | --- | --- | --- | --- | --- | --- | --- |
| **IKZF1 gene**  **Deleted**  **Non-deleted** | 16  29 | 7 9  16 13  *p*=0.542 | 6 10  21 8  ***p*=0.030** | 11 4  27 1  ***p*=0.043** | 10 6  2 26  ***p<*0.001** | 2 14  7 22  *p*=0.456 | 8 8  4 24  *p*=0.037 | 11 5  10 19  ***p*=0.035** | 14 2  11 18  ***p*=0.002** | 13 3  11 18  ***p*=0.011** | 16 0  10 19  ***p<*0.001** | 14 2  5 24  ***p<*0.001** |
| **IKZF1^Plus^ profile**  **Presence**  **Absence** | 8  37 | 2 6  21 16  *p*=0.135 | 1 7  26 11  ***p*=0.004** | 4 3  34 2  ***p*=0.024** | 5 3  7 30  ***p*=0.022** | 0 8  9 28  *p*=0.179 | 4 4  9 28  *p*=0.202 | 7 1  14 23  ***p*=0.017** | 7 1  18 19  *p*=0.059 | 7 1  17 20  *p*=0.051 | 8 0  18 19  ***p*=0.014** | 7 1  12 25  ***p*=0.006** |
| **CDKN2A/2B gene**  **Deleted**  **Non-deleted** | 12  33 | 4 8  19 14  *p*=0.189 | 7 5  20 13  *p*=1 | 8 3  30 2  *p*=0.096 | 5 7  7 26  *p*=0.254 | 1 11  8 25  *p*=0.407 | 5 7  8 25  *p*=0.285 | 9 3  12 21  ***p*=0.041** | 11 1  14 19  ***p*=0.005** | 6 6  18 15  *p*=1 | 10 2  16 17  ***p*=0.046** | 6 6  13 20  *p*=0.734 |
| **PAX5 gene**  **Deleted**  **Non-deleted** | 11  34 | 4 7  19 15  *p*=0.314 | 4 7  23 11  *p*=0.086 | 6 3  32 2  *p*=0.054 | 5 6  7 27 *p*=0.131 | 0 11  9 25  *p*=0.087 | 5 6  8 26  *p*=0.251 | 9 2  12 22  ***p*=0.013** | 8 3  17 17  *p*=0.297 | 7 4  17 17  *p*=0.503 | 10 1  16 18  ***p*=0.014** | 8 3  11 23  ***p*=0.033** |
| **EBF1 gene**  **Deleted**  **Non-deleted** | 3  42 | 1 2  22 20  *p=*0.608 | 2 1  25 17  **p**=1 | 2 0  36 5  *p*=1 | 0 3  12 30  *p*=0.553 | 0 3  9 33  *p*=1 | 2 1  11 31  *p*=0.196 | 9 1  12 23  *p*=0.592 | 1 2  24 18  *p*=0.577 | 1 2  23 19  *p*=0.592 | 2 1  24 18  *p*=1 | 1 2  18 24  *p*=1 |
| **BTG1 gene**  **Deleted**  **Non-deleted** | 6  39 | 0 6  23 16  *p=*0.009 | 4 2  23 16  *p*=1 | 4 1  34 4  *p*=0.479 | 2 4  10 29  *p*=0.650 | 0 6  9 30  *p*=0.323 | 1 5  12 27  *p*=0.656 | 2 1  19 23  *p*=0.592 | 2 4  23 16  *p*=0.383 | 2 4  22 17  *p*=0.396 | 3 3  23 16  *p*=0.686 | 1 5  18 21  *p*=0.222 |
| **RB1**  **Deleted**  **Non-deleted** | 4  41 | 2 2  21 20  *p*=1 | 3 1  24 17  *p*=0.640 | 2 0  36 5  *p*=1 | 1 3  11 30  *p*=1 | 0 4  9 32  *p*=0.569 | 2 2  11 30  *p*=0.567 | 2 2  19 22  *p*=1 | 2 2  23 18  *p*=1 | 1 3  23 18  *p*=0.326 | 3 1  23 18  *p*=0.627 | 0 4  19 22  *p*=0.126 |
| **ETV6**  **Deleted**  **Non-deleted** | 6  39 | 2 4  21 18  *p*=0.414 | 4 2  23 16  *p*=1 | 4 1  34 4  *p*=0.479 | 2 4  10 29  *p*=0.650 | 2 4  7 32  *p*=0.583 | 2 4  11 28  *p*=1 | 4 2  17 22  *p*=0.396 | 5 1  20 19  *p*=0.205 | 2 4  22 17  *p*=0.396 | 6 0  20 19  ***p*=0.032** | 4 2  15 24  *p*=0.377 |
| **JAK2**  **Deleted**  **Non-deleted** | 3  42 | 0 3  23 19  *p*=0.109 | 0 3  27 15  *p*=0.058 | 2 1  36 4  *p*=0.316 | 3 0  9 33  ***p*=0.016** | 0 3  9 33  *p*=1 | 2 1  11 31  *p*=0.196 | 3 0  18 24  *p*=0.094 | 3 0  22 20  *p*=0.242 | 3 0  21 21  *p*=0.236 | 3 0  23 19  *p*=0.252 | 3 0  16 26  *p*=0.068 |
| **PAR1 region**  **Duplicated**  **Non-duplicated** | 9  36 | 5 4  18 18  *p*=1 | 8 1  19 17  *p*=0.064 | 9 0  29 5  *p*=0.566 | 1 8  11 25  *p*=0.407 | 5 4  4 32  ***p*=0.009** | 1 8  12 24  *p*=0.249 | 4 5  17 19  *p*=1 | 7 2  18 18  0.260 | 3 6  21 15  *p*=0.267 | 5 4  21 15  *p*=1 | 4 5  15 21  *p*=1 |

* two cases remained undefined; M: Male; F: Female, L: low; H: High, R: Resistance; S: Sensitivity, P: Positive; N: Negative, HR: High risk; SR: Standard risk.
