# Supplementary material for: Genomic characterization and prognostic significance of copy number alterations in Tunisian patients with acute lymphoblastic leukemia
Source: PLoS One. 2026 Feb 3;21(2):e0340696. doi: 10.1371/journal.pone.0340696 (PMC12867238; doi:10.1371/journal.pone.0340696)
Supplement: S2 Table — (DOCX) [file pone.0340696.s002.docx]

**S2 Table. CNA status according to patient characteristics and response to treatment in the ALL Pediatric/Young Adults group (n=40).**

|  | **Total, n** | **Gender**  **M F** | **WBC count***  **<50.10^3^ >50.10^3^** | **BCR::ABL1**  **Yes No** | **Diploidy**  **Hyper Other** | **Corticoid response**  **R S** | **MRD_33**  **P N** | **MRD_63**  **P N** | **Risk classification**  **HR SR** | **Relapse**  **Yes No** | **Death**  **Yes No** |
| --- | --- | --- | --- | --- | --- | --- | --- | --- | --- | --- | --- |
| **IKZF1 gene**  **Deleted**  **Non-deleted** | 8  32 | 5 3  19 13  *p*=1 | 7 1  30 1  *p*=0.372 | 3 5  1 31  ***p=*0.021** | 1 7  7 25  *p*=1 | 5 3  6 26  ***p=*0.025** | 5 3  15 17  *p*=0.695 | 7 1  14 18  ***p*=0.046** | 4 4  4 28  ***p*=0.037** | 8 0  13 19  ***p*=0.004** | 7 1  5 27  ***p<*0.001** |
| **IKZF1^Plus^ profile**  **Presence**  **Absence** | 3  37 | 3 0  21 16  *p*=0.262 | 3 0  34 2  *p*=1 | 0 3  4 33  *p*=1 | 0 3  8 29  *p*=1 | 3 0  8 29  ***p=*0.017** | 3 0  17 20  *p*=0.231 | 2 1  19 18  *p*=1 | 1 2  7 30  *p*=0.498 | 3 0  18 19  *p*=0.233 | 3 0  9 28  ***p*=0.022** |
| **CDKN2A/2B gene**  **Deleted**  **Non-deleted** | 15  25 | 10 5  14 11  *p*=0.740 | 14 1  23 1  *p*=1 | 0 15  4 21  *p*=0.278 | 2 13  6 19  *p*=0.686 | 7 8  4 21  *p=*0.065 | 9 6  11 14  *p*=0.514 | 10 5  11 14  *p*=0.204 | 3 12  5 20  *p*=1 | 9 6  12 13  *p*=0.727 | 5 10  7 18  *p*=0.736 |
| **PAX5 gene**  **Deleted**  **Non-deleted** | 8  32 | 7 1  17 15  *p*=0.114 | 7 0  30 2  *p*=1 | 0 8  4 28 *p*=0.566 | 1 7  7 25  *p*=1 | 3 5  8 24  *p=*0.660 | 5 3  15 17  *p*=0.695 | 3 5  18 14  *p*=0.442 | 1 7  7 25  *p*=1 | 5 3  16 16  *p*=0.698 | 3 5  9 23  *p*=0.677 |
| **EBF1 gene**  **Deleted**  **Non-deleted** | 3  37 | 2 1  22 15  *p*=1 | 2 0  35 2  *p*=1 | 0 3  4 33  *p*=1 | 0 3  8 29  *p*=1 | 1 2  10 27  *p*=1 | 2 1  18 19  *p*=1 | 1 2  20 17  *p*=0.596 | 0 3  8 29  *p*=1 | 1 2  20 17  *p*=0.596 | 0 3  12 25  *p*=0.541 |
| **BTG1 gene**  **Deleted**  **Non-deleted** | 4  36 | 1 3  23 13  *p*=0.283 | 3 0  34 2  *p*=1 | 0 4  4 32  *p*=1 | 0 4  8 28  *p*=0.566 | 0 4  11 25  *p*=0.560 | 2 2  18 18  *p*=1 | 2 2  19 17  *p*=1 | 0 4  8 28  *p*=0.566 | 2 2  19 17  *p*=1 | 0 4  12 24  *p*=0.297 |
| **RB1**  **Deleted**  **Non-deleted** | 9  31 | 6 3  18 13  *p*=0.717 | 8 0  29 2  *p*=1 | 0 9  4 27  *p*=0.557 | 1 8  7 24  *p*=0.655 | 3 6  8 23  *p*=0.686 | 6 3  14 17  *p*=0.451 | 5 4  16 15  *p*=1 | 1 8  7 24  *p*=0.655 | 6 3  15 16  *p*=0.457 | 2 7  10 21  *p*=0.697 |
| **ETV6**  **Deleted**  **Non-deleted** | 4  36 | 2 2  22 14  *p*=1 | 3 0  34 2  *p*=1 | 0 4  4 32  *p*=1 | 2 2  6 30  *p*=0.172 | 0 4  11 25  *p*=0.560 | 2 2  18 18  *p*=1 | 3 1  18 18  *p*=0.607 | 0 4  8 28  *p*=0.5 66 | 4 0  17 19  *p*=0.108 | 2 2  10 26  *p*=0.570 |
| **JAK2**  **Deleted**  **Non-deleted** | 4  36 | 4 0  20 16  *p*=0.136 | 4 0  33 2  *p*=1 | 0 4  4 32  *p*=1 | 0 4  8 28  *p*=0.566 | 1 3  10 26  *p*=1 | 2 2  18 18  *p*=1 | 2 2  19 17  *p*=1 | 0 4  8 28  *p*=0.566 | 2 2  19 17  *p*=1 | 1 3  11 25  *p*=1 |
| **PAR1 region**  **Duplicated**  **Non-duplicated** | 9  31 | 5 4  19 12  *p*=1 | 9 0  28 2  *p*=1 | 1 8  3 28  *p*=1 | 5 4  3 28  ***p*=0.008** | 1 8  10 21  *p*=0.399 | 4 5  16 15  *p*=1 | 6 3  15 16  *p*=0.457 | 2 7  6 25  *p*=1 | 4 5  17 14  *p*=0.712 | 3 6  9 22  *p*=1 |

*One case remained undefined; M: Male; F: Female, L: low; H: High, R: Resistance; S: Sensitivity, P: Positive; N: Negative, HR: High risk; SR: Standard risk.
