# Supplementary material for: Genomic characterization and prognostic significance of copy number alterations in Tunisian patients with acute lymphoblastic leukemia
Source: PLoS One. 2026 Feb 3;21(2):e0340696. doi: 10.1371/journal.pone.0340696 (PMC12867238; doi:10.1371/journal.pone.0340696)
Supplement: S3 Table — (DOCX) [file pone.0340696.s003.docx]

**S3 Table. CNA status according to patient characteristics and response to treatment in the ALL Adults group (n=20).**

|  | **Total, n** | **Gender**  **M F** | **WBC count* <50.10^3^ >50.10^3^** | **BCR::ABL1**  **Yes No** | **Diploidy**  **Hyper Other** | **Corticoid response**  **R S** | **MRD_33**  **P N** | **MRD_63**  **P N** | **Risk classification**  **HR SR** | **Relapse**  **Yes No** | **Death**  **Yes No** |
| --- | --- | --- | --- | --- | --- | --- | --- | --- | --- | --- | --- |
| **IKZF1 gene**  **Deleted**  **Non-deleted** | 10  10 | 4 6  7 3  *p*=0.370 | 6 3  10 0  *p*=0.087 | 7 3  1 9  ***p*=0.020** | 1 9  1 9  *p*=1 | 3 7  2 8  *p*=1 | 8 2  1 9  ***p*=0.005** | 9 1  2 8  ***p*=0.003** | 10 0  9 1  *p*=1 | 10 0  3 7  ***p*=0.003** | 9 1  2 8  ***p*=0.005** |
| **IKZF1^Plus^ profile**  **Presence**  **Absence** | 7  13 | 1 6  10 3  ***p*=0.017** | 3 3  13 0  *p*=0.056 | 5 2  3 10  *p*=0.062 | 0 7  2 11  *p*=0.521 | 3 4  2 11  *p*=0.290 | 6 1  3 10  ***p*=0.017** | 7 0  4 9  ***p*=0.005** | 7 0  12 1  *p*=1 | 7 0  6 7  ***p*=0.044** | 6 1  5 8  *p*=0.07 |
| **CDKN2A/2B gene**  **Deleted**  **Non-deleted** | 5  15 | 1 4  10 5  *p*=0.127 | 2 2  14 1  *p*=0.097 | 5 0  3 12  ***p*=0.004** | 0 5  2 13  *p*=1 | 2 3  3 12  *p*=0.560 | 4 1  5 10  *p*=0.127 | 5 0  6 9  ***p*=0.038** | 5 0  14 1  *p*=1 | 5 0  8 7  *p*=0.114 | 4 1  7 8  *p*=0.319 |
| **PAX5 gene**  **Deleted**  **Non-deleted** | 7  13 | 1 6  10 3  ***p*=0.017** | 3 3  13 0  *p*=0.021 | 5 2  3 10  *p*=0.062 | 0 7  2 11  *p*=0.521 | 3 4  2 11  *p*=0.290 | 6 1  3 10  ***p*=0.017** | 7 0  4 9  ***p*=0.005** | 7 0  12 1  *p*=1 | 7 0  6 7  ***p*=0.044** | 6 1  5 8  *p*=0.07 |
| **EBF1 gene**  **Deleted**  **Non-deleted** | 1  19 | 0 1  11 8  *p*=0.450 | 1 0  15 3  *p*=1 | 0 1  8 11  *p*=1 | 0 1  2 17  *p*=1 | 0 1  5 14  *p*=1 | 1 0  8 11  *p*=0.450 | 1 0  10 9  *p*=1 | 1 0  18 1  *p*=1 | 1 0  12 7  *p*=1 | 1 0  10 9  *p*=1 |
| **BTG1 gene**  **Deleted**  **Non-deleted** | 4  16 | 0 4  11 5  ***p*=0.026** | 2 2  14 1  *p*=0.097 | 2 2  6 10  *p*=1 | 0 4  2 14  *p*=1 | 2 2  3 13  *p*=0.249 | 3 1  6 10  *p*=0.285 | 3 1  8 8  *p*=0.591 | 4 0  15 1  *p*=1 | 3 1  10 6  *p*=1 | 3 1  8 8  *p*=0.591 |
| **RB1**  **Deleted**  **Non-deleted** | 1  19 | 1 0  10 9  *p*=1 | 0 0  16 3  *p*=0.381 | 1 0  7 12  *p*=0.400 | 0 1  2 17  *p*=1 | 0 1  5 14  *p*=1 | 0 1  9 10  *p*=1 | 1 0  10 9  *p*=1 | 1 0  18 1  *p*=1 | 1 0  12 7  *p*=1 | 0 1  11 8  *p*=0.450 |
| **ETV6**  **Deleted**  **Non-deleted** | 2  18 | 0 2  11 7  *p*=0.189 | 1 1  15 2  *p*=0.298 | 2 0  6 12  *p*=0.147 | 0 2  2 16  *p*=1 | 2 0  3 15  *p*=0.053 | 2 0  7 11  *p*=0.189 | 2 0  9 9  *p*=0.479 | 2 0  17 1  *p*=1 | 2 0  11 7 *p*=0.527 | 2 0  9 9  *p*=0.479 |
| **JAK2**  **Deleted**  **Non-deleted** | 3  17 | 0 3  11 6  *p*=0.074 | 2 1  14 2  *p*=0.422 | 3 0  5 12  ***p*=0.049** | 0 3  2 15  *p*=1 | 1 2  4 13  *p*=1 | 3 0  6 11  *p*=0.074 | 3 0  8 9  *p*=0.218 | 3 0  16 1  *p*=1 | 3 0  10 7  *p*=0.521 | 3 0  8 9  *p*=0.218 |
| **PAR1 region**  **Duplicated**  **Non-duplicated** | 1  19 | 1 0  10 9  *p*=1 | 1 0  15 3  *p*=1 | 0 1  8 11  *p*=1 | 0 1  2 17  *p*=1 | 1 0  4 15  *p*=0.250 | 0 1  9 10  *p*=1 | 1 0  10 9  *p*=1 | 1 0  18 1  *p*=1 | 1 0  12 7  *p*=1 | 1 0  10 9  *p*=1 |

*One case remained undefined; M: Male; F: Female, L: low; H: High, R: Resistance; S: Sensitivity, P: Positive; N: Negative, HR: High risk; SR: Standard risk
