# Supplementary material for: Genomic characterization and prognostic significance of copy number alterations in Tunisian patients with acute lymphoblastic leukemia
Source: PLoS One. 2026 Feb 3;21(2):e0340696. doi: 10.1371/journal.pone.0340696 (PMC12867238; doi:10.1371/journal.pone.0340696)
Supplement: S4 Table — (DOCX) [file pone.0340696.s004.docx]

|  | **OS** | | | | **RFS** | | | |
| --- | --- | --- | --- | --- | --- | --- | --- | --- |
|  | **p value** | **HR** | **CI 95%** |  | **p value** | **HR** | **CI 95%** |  |
| **Parameters** |  | | **Low** | **High** |  | | **Low** | **High** |
| **IKZF1 deletion** | **0.009** | 6.664 | 1.603 | 27.710 | **0.021** | 4.477 | 1.253 | 15.993 |
| **Age** | 0.650 | 0.716 | 0.169 | 3.039 | 0.836 | 0.878 | 0.258 | 2.986 |
| **BCR::ABL1** | 0.107 | 0.336 | 0.090 | 1.264 | 0.203 | 0.434 | 0.120 | 1.567 |
| **Diploidy** | 0.142 | 2.710 | 0.717 | 10.248 | 0.458 | 0.683 | 0.250 | 1.868 |
| **WBC count** | 0.096 | 0.315 | 0.081 | 1.225 | 0.681 | 0.773 | 0.227 | 2.633 |
| **MRD at day 33** | **0.005** | 8.168 | 1.883 | 35.439 | **0.007** | 4.298 | 1.487 | 12.424 |
| **MRD at day 63** | **0.032** | 6.717 | 1.181 | 38.198 | **0.006** | 9.720 | 1.939 | 48.738 |
| **Initial risk stratification** | 0.064 | 5.241 | 0.906 | 30.324 | 0.226 | 2.362 | 0.587 | 9.503 |
| **Treatment Protocol** | 0.650 | 0.716 | 0.169 | 3.039 | 0.836 | 0.878 | 0.258 | 2.986 |

**S4 Table. Multivariate Cox model assessing the impact of IKZF1 deletions on survival in the B-ALL cases (n=45).**

OS: Overall survival, RFS: Relapse free survival, HR: Hazard ratio, CI 95%: confidence interval 95%
