# Supplementary material for: Genomic characterization and prognostic significance of copy number alterations in Tunisian patients with acute lymphoblastic leukemia
Source: PLoS One. 2026 Feb 3;21(2):e0340696. doi: 10.1371/journal.pone.0340696 (PMC12867238; doi:10.1371/journal.pone.0340696)
Supplement: S5 Table — (DOCX) [file pone.0340696.s005.docx]

**S5 Table. Multivariate Cox model assessing the impact of IKZF1 deletions on survival in the Pediatric/Young Adult ALL cases (n=40).**

|  | **OS** | | | | **RFS** | | | |
| --- | --- | --- | --- | --- | --- | --- | --- | --- |
|  | **p value** | **HR** | **CI 95%** |  | **p value** | **HR** | **CI 95%** |  |
| **Parameters** |  | | **Low** | **High** |  | | **Low** | **High** |
| **IKZF1 deletion** | **0.010** | 9.168 | 1.692 | 49.669 | 0.114 | 2.883 | 0.775 | 10.726 |
| **BCR::ABL1** | 0.708 | 1.543 | 0.160 | 14.912 | 0.821 | 0.800 | 0.116 | 5.521 |
| **Diploidy** | 0.036 | 6.996 | 1.133 | 43.192 | 0.749 | 0.824 | 0.252 | 2.696 |
| **WBC count** | 0.549 | 0.436 | 0.029 | 6.560 | 0.436 | 0.437 | 0.055 | 3.503 |
| **MRD at day 33** | **0.021** | 12.500 | 1.471 | 106.259 | 0.112 | 3.130 | 0.766 | 12.798 |
| **MRD at day 63** | 0.836 | 1.283 | 0.121 | 13.616 | 0.075 | 4.755 | 0.853 | 26.512 |
| **Initial risk stratification** | 0.867 | 0.873 | 0.179 | 4.269 | 0.307 | 2.073 | 0.512 | 8.395 |

OS: Overall survival, RFS: Relapse free survival, HR: Hazard ratio, CI 95%: confidence interval 95%
